# Cannabinoid CB_1_ receptor expression in oligodendrocyte progenitors of the hippocampus revealed by the NG2-EYFP-knockin mouse

**DOI:** 10.3389/fnana.2022.1030060

**Published:** 2022-10-28

**Authors:** Andrea Manterola, Juan Carlos Chara, Tania Aguado, Javier Palazuelos, Carlos Matute, Susana Mato

**Affiliations:** ^1^Department of Neurosciences, University of the Basque Country UPV/EHU, Leioa, Spain; ^2^Achucarro Basque Center for Neuroscience, Leioa, Spain; ^3^Centro de Investigación Biomédica en Red sobre Enfermedades Neurodegenerativas (CIBERNED), Madrid, Spain; ^4^Department of Biochemistry and Molecular Biology, Instituto Universitario de Investigación en Neuroquímica (IUIN), Complutense University, Madrid, Spain; ^5^Instituto Ramón y Cajal de Investigación Sanitaria (IRYCIS), Madrid, Spain; ^6^Neuroinmunology Group, Biocruces Bizkaia Health Research Institute, Barakaldo, Spain

**Keywords:** cannabinoid CB_1_ receptor, oligodendrocyte progenitor cell (OPC), NG2 glia, hippocampus, immunoelectron microscopy, NG2-EYFP reporter mice

## Abstract

Adult oligodendrocyte progenitor cells (OPCs) give rise to myelinating oligodendrocytes through life and play crucial roles in brain homeostasis and plasticity during health and disease. Cannabinoid compounds acting through CB_1_ receptors promote the proliferation and differentiation of OPCs *in vitro* and facilitate developmental myelination and myelin repair *in vivo*. However, CB_1_ receptor expression in adult OPCs *in situ* has not been corroborated by anatomical studies and the contribution of this receptor population to the (re)myelination effects of cannabinoids remains a matter of debate. Using electron microscopy methods applied to NG2-EYFP reporter mice we assessed the localization of CB_1_ receptors in OPCs of the adult mouse hippocampus. To control for the specificity of CB_1_ receptor immunostaining we generated transgenic mice bearing EYFP expression in NG2 glia and wild-type (NG2-EYFP-CB_1_^+/+^) and knockout (NG2-EYFP-CB_1_^–/–^) for CB_1_ receptors. Double immunogold and immunoperoxidase labeling for CB_1_ and EYFP, respectively, revealed that CB_1_ receptors are present in a low proportion of NG2 positive profiles within hippocampal s*tratum radiatum* of NG2-EYFP-CB_1_^+/+^ mice. Quantitative analysis of immunogold particles in synaptic structures and NG2 profiles showed that CB_1_ receptors are expressed at lower density in adult OPCs than in glutamatergic cells of the rodent hippocampus. These results highlight the presence of CB_1_ receptors in adult OPCs thus providing an anatomical substrate for the remyelination promoting effects of cannabinoids and open a novel perspective on the roles of the endocannabinoid system in brain physiology through the modulation of NG2 glia.

## Introduction

Oligodendrocyte precursor cells (OPCs), also known as NG2 glia due to their expression of proteoglycan CSPG4, are highly abundant at birth and provide myelinating oligodendrocytes during postnatal development (Nave and Werner, 2014; Dimou and Gallo, 2015). Adult OPCs persist ubiquitously and uniformly distributed throughout the central nervous system and play important homeostatic functions in health and disease (Dimou and Gallo, 2015). Accumulating evidence highlights that gray matter NG2 glia fine-tune neuronal networks in response to synaptic signals with emerging implications in behavioral responses (Sakry et al., 2014; Birey et al., 2015; Zhang et al., 2021). It is also well established that adult OPCs display a robust proliferative and migratory potential through life, giving rise to new myelin forming oligodendrocytes that enable adaptive myelination and motor skill learning as well as myelin repair in case of acute injury and, to some extents, chronic demyelinating diseases such as multiple sclerosis (MS) (Young et al., 2013; Xiao et al., 2016; Franklin and Ffrench-Constant, 2017).

Endocannabinoids are neuromodulatory lipids that regulate a myriad of brain functions through the activation of cannabinoid CB_1_ receptors heterogeneously expressed in neurons and glial cells. Endogenous and exogenous cannabinoids facilitate developmental myelination and exert myelin protective and (re)myelination promoting effects in animal models of MS, suggesting that OPCs and oligodendrocytes might be direct targets of these compounds (Bernal-Chico et al., 2015; Feliú et al., 2017; Aguado et al., 2021; Huerga-Gómez et al., 2021). This hypothesis is further supported by pharmacological assessments in culture systems showing that activation of CB_1_ receptors promotes the proliferation, migration and differentiation of OPCs (Gomez et al., 2010; Gomez et al., 2015; Sanchez-Rodriguez et al., 2018) and mediates oligodendrocyte protection from excitotoxicity (Bernal-Chico et al., 2015). The idea that oligodendroglia express CB_1_ receptors gained momentum with recent work based in single cell RNA sequencing that demonstrates the presence of *Cnr1* transcripts at multiple stages of the oligodendrocyte lineage with the highest transcript levels corresponding to newly formed oligodendrocyte populations responsive to complex motor learning (Marques et al., 2016). Further, genetic inactivation of CB_1_ receptors in OPCs disrupts postnatal myelination and leads to motor and cognitive alterations in young adult mice (Sánchez-de la Torre et al., 2022). However, anatomical studies have failed to validate the expression of these proteins in OPCs beyond anecdotal observations in *postmortem* tissue from MS patients (Benito et al., 2007). Indeed, the vast majority of CB_1_ receptors accumulate on axon terminals with expression levels in GABAergic neurons vastly outnumbering that of glutamatergic cells and astrocytes as demonstrated by using electron microscopy (EM) (Kawamura et al., 2006; Gutiérrez-Rodríguez et al., 2017; Gutiérrez-Rodríguez et al., 2018). Focusing on the role of endocannabinoids in oligodendrocyte biology, our group has recently reported the localization of CB_1_ receptors in mature oligodendrocytes of the subcortical white matter by EM (Bernal-Chico et al., 2022). Yet, the presence and relative amounts of CB_1_ receptors in OPCs have not been described due to the difficulties associated to the identification of these cells at the EM level. In this study, we investigated the ultrastructural localization of CB_1_ receptors in adult OPCs by using a double pre-embedding immunogold and immunoperoxidase method that allows for the simultaneous detection of NG2 positive profiles and CB_1_ proteins. To identify NG2 positive structures we took advantage of the NG2 knockin mouse line that expresses enhanced yellow fluorescent protein (EYFP) under the control of the *Cgsp4* gene promoter and is regarded as a good model for the study of adult OPCs (Karram et al., 2008). We focused our analysis in the hippocampus because this structure exhibits high CB_1_ receptor levels and the precise localization of the CB_1_ protein has been analyzed in detail in this brain area by using EM (Hájos et al., 2000; Kawamura et al., 2006; Gutiérrez-Rodríguez et al., 2017; Gutiérrez-Rodríguez et al., 2018). Comparative analysis of wild-type and CB_1_ null EYFP mutants unveils that CB_1_ receptors are present and quantifiable in NG2 glial cells of the adult mouse hippocampus. We hypothesize that CB_1_ receptors in adult OPCs may modulate the activity of these cells and their interactions with neuronal networks with potential implications in learning and memory processes, as well as in myelin repair.

## Materials and methods

### Ethics statement

Experiments were approved by the Committee of Ethics for Animal Welfare of the University of the Basque Country and the local competent authorities.

### Animals

For EM analyses of CB_1_ receptor localization in adult OPCs we generated mutant mice heterozygous for EYFP expression in NG2 cells and wild-type or knockout for CB_1_ receptors in all cell types, herein referred to as NG2-EYFP-CB_1_^+/+^ and NG2-EYFP-CB_1_^–/–^, respectively. The NG2-EYFP-CB_1_^–/–^ double mutants were used as controls for the specificity of CB_1_ receptor immunolabeling in their littermate NG2-EYFP-CB_1_^+/+^ mice. Experimental mice were obtained by crossing NG2-EYFP^±^ knockin mice (Karram et al., 2008) with CB_1_^±^ mice (Marsicano et al., 2002) (colony founders provided by Dr. Jacqueline Trotter and Dr. Beat Lutz at the Institute of Molecular Biology, Mainz, Germany). Animals were housed under standard conditions (12 h light/dark cycles) with access to food and water *ad libitum*.

### Tissue isolation

Mice at postnatal day 60 were intraperitoneally (i.p.) anesthetized with ketamine/xylazine (80/10 mg/Kg; Imalgene^®^, Mérial/Rompun^®^, Bayer) and transcardially perfused with saline solution (0.9% NaCl; pH 7.4) to clear blood vessels followed by fixative solution containing 4% paraformaldehyde, 0.1% glutaraldehyde and 0.2% picric acid in 0.1 M phosphate buffer (PB; pH 7.4), using a peristaltic pump. After extraction, brains were postfixed overnight in 4% paraformaldehyde at 4^°^C. Coronal sections (40 μm-thick) containing the hippocampus were obtained on a vibratome (VT1000S, Leica) and stored in 0.1 M PB containing 0.02% sodium azide until use.

### Double pre-embedding immunogold and immunoperoxidase method for electron microscopy

Coronal sections from NG2-EYFP-CB_1_^+/+^ and NG2-EYFP-CB_1_^–/–^ mice containing the hippocampus were pre-incubated in 1% hydrogen peroxide (H_2_O_2_) for 15 min at room temperature (RT). After several washes in Tris-HCl buffered saline (TBS; pH 7.4) slices were blocked in 10% BSA, 0.1% sodium azide and 0.02% saponin in TBS for 30 min at RT. Subsequently, sections were incubated with primary polyclonal rabbit anti-CB_1_ receptor antibody (1:500; ImmunoGenes) and monoclonal rat anti-GFP antibody (1:2,000; Nacalai Tesque) prepared in blocking solution with 0.004% saponin for 2 days at 4^°^C. Following several washes in 1% BSA/TBS, sections were incubated for 4 h at RT with biotinylated anti-rat IgG made in horse (1:200; Vector Laboratories, Burlingame, CA, USA) and 1.4 nm gold-labeled goat anti-rabbit IgG (1:200; Nanoprobes Inc.) prepared in 1% BSA/TBS with 0.004% saponin. Tissue slices were then washed in 1% BSA/TBS and treated with avidin-biotinylated-horseradish peroxidase complex (ABC; Elite, Vector laboratories). Sections were washed overnight in 1% BSA/TBS at 4^°^C, postfixed in 1% glutaraldehyde in TBS for 10 min at RT and washed in ddH_2_0. Gold particles were silver-intensified with a HQ Silver kit (Nanoprobes) in the dark for 12 min and tissue was washed with ddH20 followed by 0.1 M phosphate buffer (PB; pH 7.4). The immunoperoxidase was developed using in 3′diaminobenzidine (DAB) as a chromogen (Roche Diagnostics). The day after, sections were osmicated (1% OsO_4_ in 0.1 M PB; pH 7.4) for 30 min. After 3 × 10 min washes in 0.1 M PB, tissue sections were dehydrated in graded ethanol concentrations (50–100%) to propylene oxide and embedded in epoxy resin (Sigma-Aldrich) by immersion in decreasing concentration of propylene oxide (1:3 for 30 min, 1:1 for 1 h and 3:1 for 2 h). Tissue was then embedded in fresh resin overnight and allowed to polymerize at 60^°^C for 2 days. Following visualization at the light microscope, selected tissue portions were trimmed and glued onto epoxy resin capsules. Semithin sections (500 nm−thick) were cut from epoxy blocks using a Power Tome ultramicrotome (RMC Boeckeler) and stained with 1% toluidine blue. Ultrathin (50–60 nm-thick) sections were then cut with a diamond knife (Diatome), collected on nickel mesh grids and stained with 4% uranyl acetate for 30 min and 2.5% lead citrate for EM visualization.

### Semi-quantification of the CB_1_ receptor immunogold and immunoperoxidase staining

The pre-embedding immunogold and immunoperoxidase methods were simultaneously applied and repeated two times on the sections obtained from NG2-EYFP-CB_1_^+/+^ and NG2-EYFP-CB_1_^–/–^ animals. Double immunolabeling was visualized with a light microscope and portions of the CA1 *stratum radiatum* with consistent staining of CB_1_ receptors and NG2 cells were trimmed down for ultrathin sectioning. To standardize conditions and avoid false negatives, only the first 20 ultrathin sections were collected onto the grids and photographed for analysis.

Ultrathin sections were examined with a Jeol JEM 1400 Plus electron microscope at the Service of Analytical and High-Resolution Microscopy in Biomedicine of University of the Basque Country. For the analysis of CB_1_ receptor localization in OPCs, the electron micrographs were taken with a digital sCMOS camera (Hamamatsu Photonics) at magnification 8,000–15,000 X. Sampling was always carefully and accurately carried out in the same way for all the animals studied. Positive OPC processes in hippocampus *stratum radiatum* were identified by the presence of DAB immunodeposits. The density of CB_1_ receptors in excitatory and inhibitory terminals was analyzed in 20 electron micrographs per animal taken in a systematic manner at magnification of 8,000 X. Image-J software (NIH) was used to measure the membrane length (perimeter) of OPC processes and synaptic terminals. Positive labeling was considered if at least one immunoparticle was found within approximately 30 nm from the membrane. Percentages of CB_1_ receptor positive processes, as well as immunolabeling density (particles/μm membrane), were analyzed. Results correspond to the analysis of 28–94 DAB positive OPC processes, 15–24 inhibitory and 97–107 excitatory synaptic terminals per animal. Minor adjustments in contrast and brightness were made to the figures using Adobe Photoshop.

### Statistical analysis

Data are presented as mean ± SEM of 3–5 different animals. Statistical analyses were performed with using GraphPad Prism 9 for Windows (GraphPad Software Inc.) using Shapiro-Wilk normality tests followed by two-sided unpaired Student’s *t*-tests. *P*-values < 0.05 were considered statistically significant.

## Results

### Light microscopy assessment of CB_1_ receptors and OPCs in the hippocampus of NG2-EYFP-CB_1_ mutant mice

**FIGURE 1:**
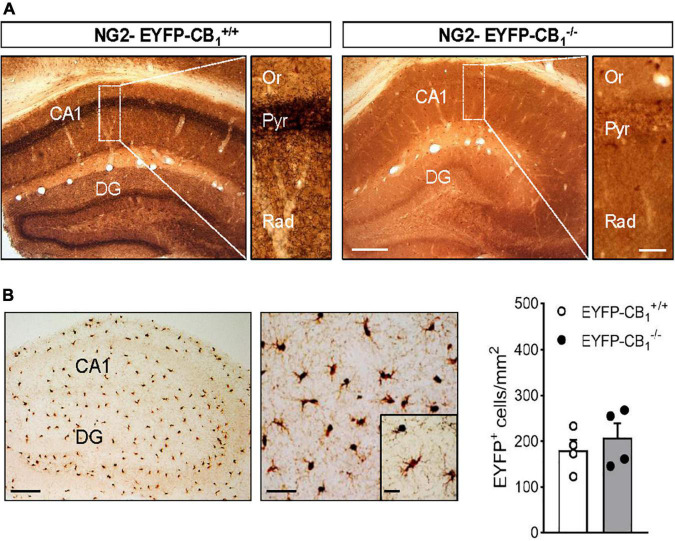
Combined immunogold and immunoperoxidase for CB_1_ and EYFP in the hippocampus of NG2-EYFP mice. **(A)** Representative images show pre-embedding silver intensified immunogold labeling of CB_1_ receptors and immunoperoxidase labeling EYFP in coronal brain sections from NG2-EYFP-CB_1_^+/+^ and NG2-EYFP-CB_1_^– /–^ mice. Intense bands of CB_1_ receptor immunostaining are observed in the *stratum pyramidale* and molecular dentate gyrus. Higher magnification images show dense CB_1_ receptor neuropil labeling throughout CA1 *stratum radiatum*. The hippocampus of a NG2-EYFP-CB_1_^– /–^ mice shows no CB_1_ immunopositive profiles and allows visualization of uniformly distributed DAB^+^ NG2 cells (white asterisks). **(B)** Immunoperoxidase labeling of EYFP in the hippocampus of NG2-EYFP^±^-CB_1_^+/+^ mutant mice showing the typical distribution and morphology of NG2 cells in this brain area. Densities of EYFP positive cells in CA1 *stratum radiatum* were statistically similar between genotypes. CA1, region 1 of *cornu ammonis*; Or, *stratum oriens*; Pyr, *stratum pyramidale*; Rad, *stratum radiatum*; DG, dentate gyrus. Scale bars: 200 and 25 μm **(A)**; 200, 50, and 25 μm **(B)**.

To study the localization of CB_1_ receptors in adult OPCs we applied a combined immunogold and immunoperoxidase method to brain tissue from NG2-EYFP-CB_1_^+/+^ and NG2-EYFP-CB_1_^–/–^ mice. In these animals, OPC processes were identified by DAB immunodeposits of EYFP and the CB_1_ receptor was detected by silver-intensified immunogold labeling. The pattern of CB_1_ receptor immunostaining was first verified in NG2-EYFP-CB_1_^+/+^ mice by light microscopy evaluation of coronal sections containing the hippocampus (Figure 1A). We detected intense immunoreactivity for CB_1_ receptors in a fibrous pattern throughout the hippocampal CA1 and CA3 subfields and the dentate gyrus (Figure 1A), in good agreement to previous studies addressing the localization of CB_1_ receptors in this brain area (Hájos et al., 2000; Gutiérrez-Rodríguez et al., 2017). The distribution of CB_1_ receptor immunostaining followed the layered structure of the hippocampus showing the highest density in the inner molecular layer of the dentate gyrus, the pyramidal cell layer and the *stratum radiatum* of the CA1 and CA3 subfields. The specificity of these signals was confirmed by their virtual disappearance in tissue sections from NG2-EYFP-CB_1_^–/–^ mice (Figure 1A). On the other hand, the overall DAB pattern in NG2-EYFP-CB_1_^+/+^ matched the known uniform distribution of NG2 cells in the rodent hippocampus (Karram et al., 2008). NG2-EYFP-CB_1_^–/–^ mice lacking CB_1_ receptor expression exhibited DAB positive cells in CA1 *stratum radiatum* similar in morphology and number to the cells in NG2-EYFP-CB_1_^+/+^ mice (Figures 1A,B).

### Electron microscopy localization of CB_1_ receptors in OPCs of the CA1 *stratum radiatum*

**FIGURE 2:**
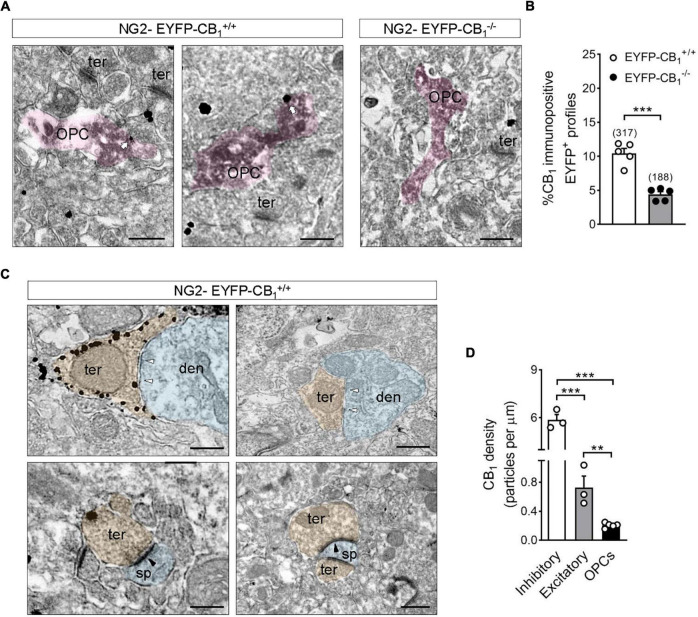
Electron microscopy localization of CB_1_ receptors in OPCs of the adult mouse hippocampus. **(A)** Representative electron micrographs showing pre-embedding silver intensified immunogold labeling of CB_1_ receptors and immunoperoxidase labeling of EYFP in coronal brain sections from adult NG2-EYFP mice. CB_1_ receptor gold particles were localized on plasma membranes of OPC processes (white arrows). Scale bars: 500 nm. **(B)** Quantification of CB_1_ receptor immunopositive OPC profiles in NG2-EYFP^±^ mutant mice. The proportion of DAB positive profiles expressing CB_1_ receptor metal particles was significantly lower in NG2-EYFP-CB_1_^– /–^ mice. The numbers in parentheses on the top of each column indicate the number of OPC processes analyzed. **(C)** Distribution of CB_1_ receptors in synaptic terminals of NG2-EYFP^±^ mice. Scale bars: 500 nm. White arrowheads, inhibitory synapses; black arrowheads, excitatory synapses; den, dendrite (blue); opc, oligodendrocyte progenitor (pink); sp, dendritic spine (blue); ter, terminal (orange). **(D)** CB_1_ receptor density (particles/μm plasma membrane) was quantified in receptor positive-OPC profiles, -inhibitory and -excitatory terminals within the *stratum radiatum* of NG2-EYFP-CB_1_^+/+^. ***p < 0.0001 inhibitory terminals vs. OPCs; ***p < 0.001 inhibitory vs. excitatory terminals; **p < 0.01 excitatory terminals vs. OPCs; Student’s *t* tests.

OPCs and their processes were identified at the EM level by the presence of electron dense DAB precipitates in their cytoplasm (Figure 2A). The percentage of CB_1_ receptor immunopositive OPC processes in CA1 *stratum radiatum* of NG2-EYFP-CB_1_^+/+^ mice was 10.5 ± 0.72% (Figure 2B) and this proportion decreased to 4.4 ± 0.40% in NG2-EYFP-CB_1_^–/–^ mice (^***^p < 0.0001; Figure 2B). Comparison between the percentages of DAB positive structures bearing immunogold label between NG2-EYFP-CB_1_^+/+^ and NG2-EYFP-CB_1_^–/–^ mice suggested that at least 6% of OPCs processes from the hippocampal *stratum radiatum* of adult mice express CB_1_ receptors. At the ultrastructural level, presynaptic inhibitory terminals making symmetric synapses with postsynaptic dendrites within CA1 *stratum radiatum* of NG2-EYFP-CB_1_^+/+^ mice were decorated with high densities of CB_1_ receptor immunoparticles (Figure 2C). As expected, much less labeling was observed in excitatory terminals identified by their typical ultrastructural features, namely, axon boutons with abundant synaptic vesicles forming asymmetric synapses with postsynaptic dendritic spines (5.866 ± 0.339 vs. 0.7264 ± 0.1583 particles/μm; ^***^p < 0.001) (Figure 2C). Thus, NG2-EYFP-CB_1_^+/+^ mice show the usual CB_1_ receptor distribution and expression in hippocampal cell types. This CB_1_ receptor distribution profile virtually disappeared in NG2-EYFP-CB_1_^–/–^ mice, corroborating the specificity of the CB_1_ receptor antibody used.

The density of CB_1_ receptor gold particles on DAB positive profiles was also analyzed and compared to values calculated for synaptic terminals of NG2-EYFP-CB_1_^+/+^ animals. CB_1_ receptor immunopositive OPC profiles of the CA1 *stratum radiatum* exhibited CB_1_ receptor metal particles at a density of 0.201 ± 0.015 particles/μm on their plasma membranes. The values of CB_1_ receptor density were slightly lower in OPCs than in excitatory terminals making asymmetric synapses within the same brain area (^**^p < 0.01) and markedly reduced when compared to inhibitory synaptic profiles (^***^p < 0.0001) (Figure 2D).

## Discussion

In this study, we provide anatomical evidence that adult OPCs of the rodent hippocampus express CB_1_ receptors by taking advantage of NG2-EYFP knockin mice. Detailed immunohistochemical analysis of NG2-EYFP^±^ mice has demonstrated that almost all EYFP expressing cells express markers for oligodendrocyte precursors such as Olig2 and Sox10 at late postnatal stages while lacking proteins that exclusively mark mature oligodendrocytes, neurons, astrocytes or microglial cells (Karram et al., 2008).

A combined pre-embedding immunogold and immunoperoxidase method applied to NG2-EYFP-CB_1_^+/+^ and NG2-EYFP-CB_1_^–/–^ mice showed that at least 6% of OPC profiles within hippocampal *stratum radiatum* express CB_1_ receptors in their plasma membrane at a density of ∼0.2 particles/μm. The EM approach used in this study does not allow the 3D visualization of the complex arborization of NG2 cells and quantitative analysis of the population of OPCs that express CB_1_ receptors in a given brain area remains an issue for future analysis. On the other hand, CB_1_ receptor immunoparticle densities in receptor-positive inhibitory and excitatory terminals of the NG2-EYFP-CB_1_^+/+^ mutants were in line with previous hippocampal data in wild-type mice (Kawamura et al., 2006; Gutiérrez-Rodríguez et al., 2017). According to these previous reports using high sensitive pre-embedding immunoelectron microscopy, the proportion of CB_1_ receptor immunopositive inhibitory terminals in CA1 *stratum radiatum* is around 80% whereas only 20% of excitatory synaptic profiles express CB_1_ receptors in this brain area. Moreover, the CB_1_ receptor was present at a density of ∼4.5 particles/μm in GABAergic terminals of the CA1 *stratum radiatum* and only of ∼0.45 particles/μm in excitatory terminals within the same area (Gutiérrez-Rodríguez et al., 2017). In this study, we were able to detect CB_1_ receptor expressed in GABAergic and glutamatergic terminals at densities of 5.9 and 0.7 particles/μm, respectively. This observation shows that our immunostaining procedure detects CB_1_ receptors at the EM level with high sensitivity. Furthermore, our comparative analysis of CB_1_ receptor gold particles in synaptic compartments and NG2^+^ profiles of the same animals allows us to conclude that CB_1_ receptors are present at lower density in OPCs than in glutamatergic cells of the rodent hippocampus. It is worth mentioning, however, that CB_1_ receptor density values in OPCs are in line with previous reports addressing the expression of the receptor protein in other glial cell types, namely astrocytes. In this regard, a recent study determined that around 40% of astrocytes in hippocampal CA1 *stratum radiatum* express CB_1_ receptors at a density of 0.1–0.15 particles/μm in wild-type mice (Gutiérrez-Rodríguez et al., 2018).

In the context of previous work, the expression of CB_1_ receptors in OPCs profiles of the adult mouse brain seems low in terms of both proportion and density. However, research in the endocannabinoid field has consistently demonstrated that the physiological relevance of CB_1_ receptor functions does not correlate with the expression levels of the protein. In this regard, several pharmacological actions of (endo)cannabinoids rely on the activation of CB_1_ receptors present at low levels in forebrain glutamatergic terminals. Indeed, analysis of mutant mice lacking CB_1_ expression in cortical glutamatergic neurons and viral deletion strategies have demonstrated a relevant role of this receptor population in mediating neuroprotection (Monory et al., 2006), motor impairment and hypothermia (Monory et al., 2007), fear memories (Metna-Laurent et al., 2012), stress-induced social alterations (Dubreucq et al., 2012), and anxiety (Rey et al., 2012). In the same regard, CB_1_ receptors in hippocampal astrocytic processes have been crucially involved in synaptic plasticity and memory formation (Navarrete and Araque, 2010; Han et al., 2012; Robin et al., 2018) while being the protein density in this cellular compartment even lower than in glutamatergic synapses within the same area. Finally, low CB_1_ protein levels located at mitochondrial membranes control cellular metabolism with important implications in memory formation and social interaction (Hebert-Chatelain et al., 2016; Jimenez-Blasco et al., 2020).

The hippocampus is a key brain region relevant for information processing and has a crucial role in the encoding, consolidation and retrieval of memories. The main psychoactive cannabinoid Δ^9^-tetrahydrocannabinol (Δ^9^-THC) impairs cognitive function in humans and affects emotional and non-emotional memories in rodents by targeting the hippocampus (Puighermanal et al., 2009; Chen et al., 2013; Han et al., 2019). At present, the complex effects of (endo)cannabinoids on hippocampal-dependent behaviors are attributed to a variety of molecular mechanisms that involve the activation of CB_1_ receptors in neurons and astroglial cells. However, NG2 glia are nowadays increasingly regarded as active participants in information processing that fine-tune memory performance and behavioral responses to stress beyond their role in the generation of myelinating oligodendrocytes. Using a combination of selective complementary approaches recent studies highlight that NG2 glial cells modulate synaptic activity in the adult hippocampus and engage anxiety-like behavior *in vivo* (Birey et al., 2015; Zhang et al., 2021). In this context, our present observations point to the possibility that endogenous and exogenous cannabinoids modulate hippocampal-dependent behaviors at least in part through the activation of CB_1_ receptors expressed in adult OPCs. Future studies should focus on the characterization of CB_1_ receptor distribution in OPC populations within additional brain areas, and address the involvement of these receptor subsets in myelin formation, maintenance and repair using oligodendrocyte-specific transgenic mice.

## Data availability statement

The raw data supporting the conclusions of this article will be made available by the authors, without undue reservation.

## Ethics statement

This animal study was reviewed and approved by Committee of Ethics for Animal Welfare of the University of the Basque Country.

## Author contributions

AM participated in the experimental design, conducted, and analyzed the experiments. JCC participated in sample preparation and conducted experiments. TA and JP provided conceptual ideas and revised the manuscript. CM participated in the experimental design, analyzed the experiments, funded the project, and revised the manuscript. SM conceptualized and supervised the study, performed experimental design, funded the project, and wrote the manuscript. All authors approved the final version of the manuscript.
